# Social support and quality of life in Chinese heart transplant recipients: mediation through uncertainty in illness and moderation by psychological resilience

**DOI:** 10.3389/fpsyt.2025.1637110

**Published:** 2025-07-22

**Authors:** Chan Gao, Song Gui, Lijun Zhu, Xiaoqian Bian, Heyong Shen, Can Jiao

**Affiliations:** ^1^ School of Psychology, Shenzhen University, Shenzhen, China; ^2^ The Shenzhen Humanities & Social Sciences Key Research Bases, Center for Mental Health, Shenzhen University, Shenzhen, China; ^3^ Institute of Analytical Psychology, City university of Macau, Macau, China

**Keywords:** heart transplantation, social support, uncertainty in illness, psychological resilience, quality of life, moderated mediation model

## Abstract

**Introduction:**

In China’s collectivist healthcare context, the mechanisms linking social support to quality of life (QoL) in heart transplant recipients remain unclear. This study integrates Mishel’s uncertainty in illness theory and Confucian resilience frameworks to cross-sectionally examine dual pathways: direct enhancement of QoL through social support and indirect reduction of uncertainty in illness, moderated by culturally embedded psychological resilience.

**Methods:**

A nationwide cross-sectional study included 428 Chinese heart transplant recipients. Social support (SSRS), uncertainty in illness (MUIS-A), resilience (CD-RISC), and QoL (SF-36) were assessed. Mediation (PROCESS Model 4) and moderated mediation (Model 14) were tested using 5,000 bootstrap resamples, controlling for age, gender, and transplant duration.

**Results:**

Social support directly improved QoL (*B* = 0.625, *p* < 0.001, direct effect = 0.435, 95% CI [0.285, 0.584]) and indirectly reduced uncertainty in illness (indirect effect = 0.19, 95% CI [0.126, 0.265]). Psychological resilience moderated the uncertainty in illness-QoL link (*B* = 0.007, *p* < 0.001), with stronger negative effects in low-resilience individuals (*B* = -0.372 vs. high-resilience *B* = -0.111).

**Conclusion:**

Based on this cross-sectional study, social support demonstrates significant associations with dual pathways: directly associated with improved QoL through relational support networks and indirectly linked to reduced uncertainty in illness via culturally mediated cognitive reframing. Culturally interventions integrating family-centered care and resilience training are recommended to improve long-term outcomes.

## 1 Introduction

### 1.1 Quality of life deficits in heart transplant recipients

Heart transplantation, while life-saving for end-stage cardiac patients, introduces profound psychosocial challenges that extend far beyond surgical recovery (1–4). Mishel’s Uncertainty in Illness Theory provides a critical lens for understanding these challenges, framing uncertainty as a multidimensional stressor arising from unpredictability of symptoms, ambiguous prognoses, and the lifelong complexity of post-transplant care (5). For heart transplant recipients, this uncertainty is amplified by the delicate balance of immunosuppression, graft acceptance risks, and existential anxieties about mortality (6, 7). Empirical studies consistently link uncertainty in illness to diminished quality of life (QoL) across chronic conditions, particularly in transplant populations where medical ambiguity persists despite clinical advancements (5, 8, 9).

Complementing this perspective, Connor-Davidson’s Resilience Framework shifts focus to individual adaptability, emphasizing the capacity to reframe adversity and maintain psychological equilibrium (10). Resilience has been associated with improved health outcomes in transplant recipients, yet its interplay with uncertainty remains underexplored (10, 11). While Mishel’s theory highlights environmental buffers like social support (12), resilience models prioritize intrapsychic processes (8, 10), creating a theoretical divide that obscures their synergistic potential. This study combines existing theories to explore how psychosocial resilience, such as social support and uncertainty in illness, influence quality of life in transplant populations.

### 1.2 Social support and quality of life

Empirical evidence establishes social support as a significant predictor of QoL in transplant populations (13), operating through two empirically validated dimensions: instrumental support (tangible assistance) and emotional support (affective reassurance). Previous studies demonstrates that perceived social support directly enhances physical recovery, emotional stability, and social reintegration, thereby improving overall QoL (8, 14). Instrumental support improves physical functioning in transplant recipients. This includes assistance with medication management (15). Instrumental support also encompasses help coordinating medical appointments (4). Concurrently, emotional support dimensions such as distress reduction interventions (16) and companionship provision (17) associate with improved psychological well-being indices.

In China’s collectivist cultural context, this support is operationalized through filial piety, where families assume caregiving roles as a moral obligation, providing both instrumental aid (e.g., managing complex medication regimens) and emotional reassurance (18, 19). Empirical studies in Chinese chronic illness populations highlight that strong family support is associated with significantly higher QoL scores compared to isolated individuals (20, 21). This contrasts with Western models, where professional healthcare systems and individualized coping strategies dominate (22, 23), underscoring cultural specificity in support mechanisms.

### 1.3 The mediating role of uncertainty in illness

Building on Mishel’s foundational work (5), uncertainty in illness emerges as a critical mediating mechanism linking social support to QoL in medically complex populations (8). Grounded in Mishel’s Uncertainty in Illness Theory, this construct is defined as the inability to determine the meaning of illness-related events, characterized by perceptions of ambiguity regarding symptoms, unpredictability of disease progression, complexity of treatment regimens, and lack of adequate information. Within the context of heart transplantation, patients face sustained uncertainty stemming from graft viability, immunosuppression management, risk of rejection, and long-term prognosis (1, 6). Empirical evidence across diverse chronic illness populations consistently demonstrates that elevated illness uncertainty correlates strongly with diminished quality of life outcomes (5, 9, 24). Research further suggests that social support may influence quality of life partly through its capacity to mitigate perceptions of illness-related uncertainty (8, 25).

Supportive interactions can provide informational clarity, enhance predictability, and reduce the perceived complexity of illness management, thereby potentially decreasing uncertainty levels (16, 26). For instance, investigations in hemodialysis and heart failure cohorts report inverse associations between perceived social support and illness uncertainty, with evidence suggesting uncertainty functions as an intermediary pathway linking social resources to well-being (15, 27).

However, the specific mediating role of uncertainty in illness within the relationship between social support and quality of life remains quantitatively underexamined in heart transplant populations (20, 28, 29). This gap is notable given the unique and persistent uncertainties inherent in post-transplant survivorship.

Consequently, this study quantitatively examines whether uncertainty in illness mediates the association between social support and quality of life in Chinese heart transplant recipients. Specifically, we investigate the extent to which social support influences quality of life indirectly by reducing perceptions of illness uncertainty. Verifying this mediating pathway is crucial for understanding the psychological mechanisms through which social resources operate and for identifying uncertainty reduction as a potential target for psychosocial interventions aimed at improving long-term adjustment in this population.

### 1.4 The moderating role of psychological resilience

The potential moderating role of psychological resilience in mitigating the adverse effects of uncertainty in illness on QoL warrants further investigation, particularly within specific clinical populations such as heart transplant recipients. Grounded in Connor and Davidson’s Resilience Framework (10), psychological resilience is conceptualized as the individual’s capacity to adapt positively to adversity, maintain psychological equilibrium, and effectively utilize coping resources. Emerging empirical evidence suggests resilience may function as a protective buffer. Studies in chronic illness populations indicate that individuals scoring higher on standardized resilience measures demonstrate greater tolerance for illness-related ambiguities and exhibit attenuated negative psychological responses to unpredictable health trajectories compared to those with lower resilience (30). Qualitative investigations with transplant recipients also report associations between resilience and adaptive cognitive appraisals of uncertainty (21, 31).

However, the specific moderating effect of resilience on the relationship between uncertainty in illness and quality of life remains quantitatively underexplored, especially within the context of heart transplantation. This gap is significant given the persistent and multifaceted uncertainties inherent in post-transplant management. Consequently, this study specifically examines whether psychological resilience attenuates the negative impact of uncertainty in illness on quality of life.

We hypothesize that higher levels of psychological resilience will weaken the strength of the negative association between uncertainty in illness and quality of life, meaning the detrimental effect of uncertainty will be less pronounced among individuals with greater resilience. This hypothesis aligns with the core proposition of resilience frameworks that individual differences in adaptive capacity significantly influence how stressors translate into health outcomes (10). Quantifying this moderating effect is crucial for identifying resilience as a potential intervention target to improve quality of life outcomes when uncertainty is unavoidable.

### 1.5 Confucian resilience in heart transplantation: family support, illness uncertainty, and quality of life

China’s collectivist societal structure, governed by Confucian relational ethics, fundamentally reconfigures psychosocial adaptation pathways for heart transplantation recipients (32, 33). Within this framework, familial obligation through filial piety operates as the primary support mechanism, where kin networks assume integrated caregiving roles—blending instrumental aid (e.g., medication supervision, appointment coordination) and emotional reassurance into a binding moral duty (19, 21). Concurrently, psychological resilience manifests as collective endurance, cultivated through intergenerational narratives that reframe health adversity as shared familial challenges rather than individual burdens (20, 34).

These cultural dynamics necessitate modifications to standard psychosocial models. First, social support reduces illness uncertainty through familial health brokering: relatives act as cultural translators who decode medical complexity into actionable knowledge using kinship-based decision hierarchies, thereby enhancing informational clarity (21, 26). Second, Confucian resilience moderates uncertainty’s impact by invoking familial duty framing—where health threats are reinterpreted as opportunities to fulfill generational obligations—thus attenuating distress through meaning-centered coping (31, 35).

**Figure 1:**
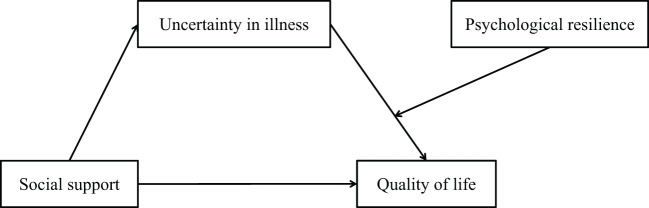
Overview of the proposed moderated mediation model.

Consequently, we propose to investigate the hypotheses (section 1.3 & 1.4) in the context of Chinese heart transplant recipients. Empirically, this model diverges from Western paradigms where uncertainty management relies on patient autonomy and professional guidance (22, 23), highlighting the need for culture-specific intervention frameworks in China’s family-centric healthcare ecosystem. The hypothesized relationships among social support, uncertainty in illness, psychological resilience, and QoL in the context of Chinese heart transplant recipients are summarized in **Figure 1**.

Proposed hypotheses:

H1: Uncertainty in illness will mediate the association between social support and quality of life within the heart transplant recipients.
H2: Psychological resilience will moderate the relationship between uncertainty in illness and quality of life within heart transplant recipients.

## 2 Materials and methods

### 2.1 Participants

Initially 440 samples collected. After random responses and missing values were excluded, 428 valid samples were finally received with a validity rate of 97.3%. Descriptive statistics for demographic variables are presented in **Table 1**. Most participants were male (75.2%, n = 322), and the age distribution was as follows: 52.6% (n = 225) were categorized as young adults (19–44 years), 36.9% (n = 158) as middle-aged adults (45–59 years), and 10.5% (n = 45) as older adults (60–66 years). Regarding educational attainment, 28.0% (n = 120) had a junior high school education or below, 26.4% (n = 113) completed high school or vocational school, 12.0% (n = 51) held an associate degree, 31.5% (n = 135) had an undergraduate degree, and 2.1% (n = 9) held a master’s degree or higher. Most participants were married (75.0%, n = 321), while 14.0% (n = 60) were unmarried and 11.0% (n = 47) were divorced. For heart transplant duration, 67.7% (n = 290) of participants received their transplant 1–5 years prior to the study, followed by 25.0% (n = 107) at 6–10 years, 5.4% (n = 23) at 11–17 years, and 1.9% (n = 8) less than a year ago.

**Table 1:** Demographic characteristics of the study.

Characteristic	Category	Frequency	Percentage
Gender	Male	322	75.2
	Female	106	24.8
Age	19-44	225	52.6
	45-59	158	36.9
	60-66	45	10.5
Education	Junior High or below	120	28.0
	High school/vocational	113	26.4
	Associate degree	51	12
	Undergraduate degree	135	31.5
	Master’s or higher	9	2.1
Marital Status	Married	321	75
	Unmarried	60	14
	Divorced	47	11
Heart Transplant Duration	Less than a year	8	1.9
	1-5	290	67.7
	6-10	107	25
	11-17	23	5.4

### 2.2 Measures

Quality of Life (QoL) was assessed using the Chinese version of the 36-item Short Form Health Survey (SF-36; 36). This instrument measures two primary domains: physical health (Physical Functioning, Role-Physical, Bodily Pain, General Health) and mental health (Vitality, Social Functioning, Role-Emotional, Mental Health). Raw scores were converted to a 0–100 scale following standard protocols, with higher scores indicating better QoL. The Cronbach’s α coefficient in this study was 0.916. Detailed item descriptions are provided in Supplementary section 1.1. Quality of Life.

Social Support was quantified using the Social Support Rating Scale (SSRS; 37), a 10-item tool widely applied in Chinese health research. It comprises three dimensions: objective support (tangible assistance), subjective support (perceived availability), and support utilization (help-seeking behaviors). Total scores range from 12 to 66, with higher values indicating stronger support networks. The Cronbach’s α coefficient for this measure was 0.92. Item-level psychometrics are reported in Supplementary section 1.2. Social Support.

Uncertainty in Illness was evaluated via the Chinese adaptation of the Mishel Uncertainty in Illness Scale for Adults (MUIS-A; 5, 38). This 32-item instrument (excluding item 15) measures four dimensions: Ambiguity, Complexity, Lack of Information, and Unpredictability. Responses were recorded on a 5-point Likert scale (1 = strongly disagree to 5 = strongly agree), with total scores ranging from 32 to 160. Higher scores reflect greater illness-related uncertainty. The Cronbach’s α coefficient in this study was 0.963. A complete item list and scoring details are archived in Supplementary section 1.3. Uncertainty in Illness.

Psychological Resilience was measured using the 25-item Connor-Davidson Resilience Scale (CD-RISC; 10), validated in Chinese populations (11). This scale comprises 25 items measuring resilience across three dimensions: Toughness, Strength, and Optimism. Responses were recorded on a 5-point Likert scale (0 = never to 4 = almost always), with total scores ranging from 0 to 100. Higher scores indicated greater resilience. The Cronbach’s α coefficient for this scale was 0.948. Subscale structures and sample items are available in Supplementary section 1.4. Psychological Resilience.

### 2.3 Procedures

The study was conducted across three high-volume cardiac transplant centers located in northern, central, and southern provinces of China. Data collection utilized a secure electronic questionnaire platform. Following institutional ethics approval from the Ethics Committee of Medical School, Shenzhen University (Approval No. PN-20250097), eligible participants were invited to complete the survey after providing digital informed consent via a certified authentication process.

Inclusion criteria comprised: (1) adults aged ≥ 18 years; (2) survival > 1-month post-transplant with successful hospital discharge; (3) intact cognitive function and communication capacity; (4) voluntary provision of informed consent. Exclusion criteria included: (1) cognitive impairment (assessed via a mental status examination score < 24); (2) severe comorbidities; (3) communication barriers (e.g., aphasia, hearing/visual impairments).

All responses were anonymized through unique participant codes, with personal identifiers permanently excluded. Real-time data encryption was implemented during transmission, and encrypted datasets were securely stored on password-protected servers. Physical copies of data were permanently destroyed post-analysis. Participants were explicitly informed of their right to withdraw at any stage, and confidentiality protocols were rigorously enforced throughout the study.

### 2.4 Statistical analyses

The data analysis was performed using SPSS 27.0 (39) following a three-stage analytical procedure. First, a common method bias test was conducted to assess potential measurement bias. Subsequently, descriptive statistics and Pearson correlation analyses were performed to examine the distribution characteristics and bivariate relationships among variables. Finally, the PROCESS macro (version 3.5) developed by Hayes (40) was employed to test mediation and moderated mediation effects. Specifically, Model 4 (simple mediation) and Model 14 (moderated mediation) were executed using the bias-corrected percentile Bootstrap method with 5,000 resamples to determine the significance of indirect effects. The bootstrapping approach we employed does not require normality assumptions for valid inference, as it generates bias-corrected confidence intervals that are robust to non-normality and other distributional issues (41, 42).

## 3 Results

### 3.1 Common method bias test

To assess common method bias, Harman’s single-factor test was performed through exploratory factor analysis (EFA) on the 106 items across four measurement scales. The results revealed 18 factors with eigenvalues exceeding 1.0 based on Kaiser’s criterion. Notably, the first unrotated factor accounted for 19.287% of the total variance explained, which is substantially below the critical threshold of 40% recommended by Podsakoff et al. (43) (43). These findings indicate no substantial common method bias in the current dataset, thereby supporting the validity of subsequent analytical procedures.

### 3.2 Descriptive statistics and correlation analyses

The descriptive statistics and bivariate correlations among key variables are presented in **Table 2**. Significant associations were observed between psychological constructs: Social support demonstrated a significant negative correlation with uncertainty in illness (*r* = -0.359, *p* < 0.001) and a positive correlation with quality of life (*r* = 0.382, *p* < 0.001). Furthermore, uncertainty in illness showed a strong inverse relationship with quality of life (*r* = -0.426, *p* < 0.001). Regarding demographic characteristics, three covariates – gender (coded 0 = male, 1 = female), age (in years), and time since heart transplantation (years post-operation) – exhibited statistically significant associations with QoL outcomes (all p-values < 0.05). These demographic variables were consequently included as covariates in subsequent multivariate analyses to control for potential confounding effects.

**Table 2:** Descriptive statistics and correlation analyses.

Variables	*M*	*SD*	1	2	3	4	5	6	7
1 Gender	–	–	1						
2 Age	44.290	11.001	-0.054	1					
3 Heart transplantation years	4.421	3.384	0.081	0.035	1				
4 Social support	35.949	10.249	-0.068	-0.025	0.072	1			
5 Uncertainty in illness	95.928	23.956	0.051	-0.093	-0.071	-0.359^***^	1		
6 Psychological resilience	52.290	18.429	-0.001	-0.051	0.017	0.078	0.088	1	
7 Quality of Life	48.595	17.371	-0.098^*^	0.104^*^	0.167^**^	0.382^***^	-0.426^***^	0.269^***^	1

*N* = 428. ^*^ *p* < 0.05, ^**^ *p* < 0.01, ^***^ *p* < 0.001. Gender: 1, male; 2, female.

### 3.3 Mediating effect test

The mediation analysis was conducted using Model 4 from PROCESS macro (version 3.5) for SPSS (39, 40), with gender, age, and years of post-heart transplantation specified as demographic covariates. As shown in **Tables 3**, **4**, social support demonstrated a significant positive predictive effect on quality of life (*B* = 0.625, *t* = 8.342, *p* < 0.001). When introducing uncertainty in illness as the mediator, while the direct effect of social support on quality of life remained statistically significant, its effect size showed substantial reduction (*B* = 0.435, *t* = 5.718, *p* < 0.001). Concurrently, uncertainty in illness exhibited a significant negative predictive effect on quality of life (*B* = -0.228, *t* = -6.985, *p* < 0.001), supporting Hypothesis H2. The analysis further confirmed that social support significantly predicted lower levels of uncertainty in illness (*B* = -0.835, *t* = -7.882, *p* < 0.001), thereby validating Hypothesis H1.

**Table 3:** Mediated model test for uncertainty in illness.

Regression equation		Fitting indicator		Coefficient significance	
Outcome variables	Predictor variables	*R²*	*F*	*B*	*t*
Quality of Life	control variables	0.183	23.717***	/	/
	Social support			0.625***	8.342
Uncertainty in illness	control variables	0.142	17.492***	/	/
	Social support			-0.835***	-7.882
Quality of Life	control variables	0.268	30.876***	/	/
	Social support			0.435***	5.718
	Uncertainty in illness			-0.228***	-6.985

*N* = 428. ***p < 0.001.

**Table 4:** Decomposition of total effect, mediated effect and direct effect.

Effect	Effect value	Boot SE	Bootstrap 95% CI	Effect ratio
Total Effect	0.625	0.075	[0.478, 0.772]	
Direct Effect	0.435	0.076	[0.285, 0.584]	69.6%
Indirect Effect	0.190	0.036	[0.126, 0.265]	30.4%

The bias-corrected bootstrap 95% confidence intervals for both the direct effect of social support and the indirect effect through uncertainty in illness excluded zero, as detailed in **Table 4**. This pattern confirms the presence of statistically significant partial mediation, consistent with Hypothesis H1.

**Figure 2:**
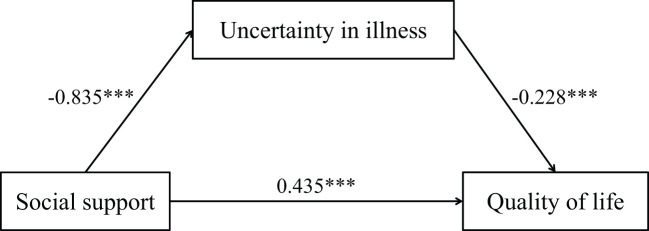
Illustration of the mediation effect of uncertainty in illness in the relationship between social support and quality of life. ***p<0.001.

Collectively, these findings substantiate that: (1) social support directly enhances quality of life, (2) uncertainty in illness negatively impacts quality of life, and (3) social support exerts indirect effects on quality of life through reducing uncertainty in illness. The complete mediation model is schematically represented in **Figure 2**.

### 3.4 Moderating effect test

The moderated mediation analysis was performed using Model 14 PROCESS macro (version 3.5) in SPSS (39, 40), incorporating gender, age, and post-transplantation duration as covariates. All continuous predictors were mean centered prior to analysis to facilitate interaction interpretation. As presented in **Tables 5**, **6**, the interaction term between Uncertainty in Illness and Psychological Resilience significantly predicted Quality of Life (*B* = 0.625, *t* = 8.342, *p* <.001), indicating a statistically significant moderating effect of psychological resilience on the uncertainty in illness-quality of life relationship, thereby confirming Hypothesis H2.

**Table 5:** Moderating effect test.

Regression equation		Fitting indicator		Coefficient significance	
Outcome variables	Predictor variables	*R²*	*F*	*B*	*t*
Quality of Life	control variables	0.377	36.368	/	/
	Social support			0.384***	5.425
	Uncertainty in illness			-0.242***	-7.923
	Psychological resilience			0.255***	6.923
	Uncertainty in illness * Psychological resilience			0.007***	4.537

Uncertainty in illness * Psychological resilience: interaction term between Uncertainty in illness and Psychological resilience. ***p < 0.001.

**Table 6:** Moderating effect of psychological resilience.

Level of moderating variable	Regression coefficient	Standard error	*t*	*p*	95% CI
Low level (-1SD)	-0.372	0.040	-9.326	<0.001	[-0.451, -0.294]
High level (+1SD)	-0.111	0.044	-2.533	0.012	[-0.197, -0.025]

**Figure 3:**
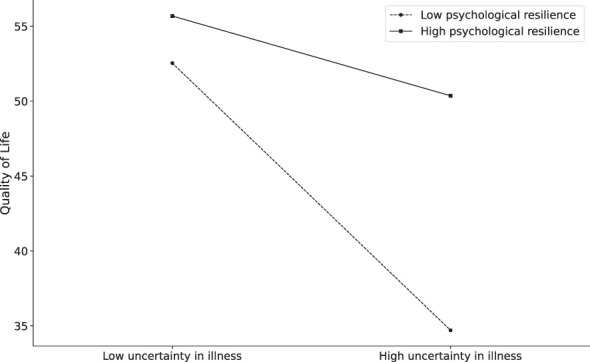
Psychological resilience moderates the relationship between Uncertainty in illness and Quality of life.

To elucidate the nature of this moderation, simple slope analysis was conducted at two conditional levels of the moderator: mean ±1 SD of Psychological Resilience (**Figure 3**). For individuals with low resilience levels (M -1 SD), uncertainty in illness demonstrated a strong negative association with quality of life (simple slope = -0.372, *t* = -9.326, *p <*0.001). Conversely, among high-resilience individuals (M +1 SD), this detrimental effect was substantially attenuated (simple slope = -0.111, *t* = -2.533, *p* < 0.05). The Johnson-Neyman technique further revealed that the conditional effect became nonsignificant (*p* > 0.05) when Psychological Resilience scores exceeded 1.8 SD above the mean (44, 45).

These findings collectively demonstrate that Psychological Resilience serves as a protective buffer, significantly mitigating the negative impact of uncertainty in illness on Quality of Life.

## 4 Discussion

This study identifies dual pathways linking social support to QoL in Chinese heart transplant recipients: a direct positive effect (*B* = 0.625, *p* < 0.001) and an indirect effect mediated through reduced illness uncertainty (indirect effect = 0.19, 95% CI [0.126, 0.265]). Critically, psychological resilience moderated the illness uncertainty-QoL relationship (interaction *B* = 0.007, *p* < 0.001). A quantifiable resilience threshold (≥ M + 1.8 SD) was identified, beyond which high resilience neutralized uncertainty’s negative impact on QoL (simple slope = -0.111 for high resilience, *p* = 0.012 vs. -0.372 for low resilience, *p* < 0.001).

These mechanisms operate within China’s collectivist context. Family systems reduce illness uncertainty through structured information mediation: relatives systematically translate clinical complexities using kinship-based decision protocols. This process enhances informational clarity, operationalizing social support’s uncertainty-reducing function observed in Chinese chronic illness populations (21, 46). Psychological resilience attenuates illness uncertainty’s impact through kinship-mediated coping processes rather than individual adaptive traits (47). This positions extended families as primary uncertainty regulators, with resilience operating as a socially contingent capacity distinct from Western autonomy-oriented coping models (10, 48).

### 4.1 The mediation effect of uncertainty in illness

Social support improves QoL by reducing uncertainty in illness. This mediating pathway represents a core psychosocial mechanism in transplant recovery. Our findings align with empirical studies of Chinese chronic illness populations demonstrating primary role of family systems in care provision (32, 46). This mediation process operates through observable behavioral patterns: kin networks integrate biomedical information using structured family decision-making processes (49). This translates health threats into collective responsibilities and reinforces treatment adherence through established norms of filial duty (21). Consequently, patients with strong family support exhibit significantly lower illness uncertainty when facing ambiguous prognoses (13), underscoring the effectiveness of this culturally embedded mechanism.

This family-centered pathway differs significantly from autonomy-focused Western models, where uncertainty management primarily occurs within individual-clinician partnerships (26). Our findings show a clear difference: multi-generational family involvement provides strong protection against medical uncertainty for patients. This protective effect is distinct and much less commonly observed in individualistic societies. Evidence supports this cultural contrast: studies show that when family support weakens, patients’ uncertainty levels rise significantly. In fact, this increase brings uncertainty up to levels typically seen in Western patient populations (50). Conversely, robust family networks offer substantially greater resilience against unpredictable health outcomes (51, 52). These empirically measured differences highlight the central role of families as active care coordinators who reshape the experience of illness (34), a function less emphasized in Western models prioritizing patient autonomy.

Clinically addressing illness uncertainty necessitates interventions that strategically engage families as cultural resources. Healthcare providers should train family members to communicate complex medical information using culturally resonant frameworks. These frameworks should be grounded in Confucian ethics (53, 54). This training involves two primary approaches. First, clinicians should guide families to reframe post-transplant complications using concepts like familial guardianship, thereby transforming biological threats into shared responsibilities (55). Second, treatment regimens should be interpreted through intergenerational commitment paradigms, explicitly linking clinical adherence to expressions of filial devotion (56). Collectively, this culturally adapted methodology reprocesses biomedical information via kinship-based cognition, converting illness uncertainty into actionable familial obligations (57).

### 4.2 The moderation effect of psychological resilience

Psychological resilience significantly moderated the illness uncertainty-QoL relationship (*B* = 0.007, *p* < 0.001). Simple slope analysis revealed a critical divergence: uncertainty strongly predicted reduced QoL in low-resilience recipients (*B* = -0.372, *p* < 0.001), but this effect attenuated markedly in high-resilience individuals (*B* = -0.111, *p* = 0.012). Crucially, resilience operated through culturally distinct pathways—specifically, recipients reframed health threats as opportunities to fulfill Confucian-based duties (e.g., enduring hardship for familial harmony) (47, 58). This collective coping mechanism fundamentally diverged from individual-focused resilience models (10).

The Johnson-Neyman technique quantified a significant threshold: uncertainty’s detrimental impact became non-significant (*p* > 0.05) when resilience exceeded 1.8 SD above the mean—indicating where collectivist cognitive processes effectively neutralize medical ambiguity. The result suggests possibility to implement intervention in clinical practices for heart transplantation recipients.

### 4.3 Limitations and future directions

While this study advances understanding of psychosocial pathways in Chinese heart transplant recipients, several limitations warrant attention. First, the cross-sectional design precludes causal inferences between variables. While the hypothesized mediation and moderation models align with theoretical frameworks, temporal dynamics (e.g., whether resilience develops after confronting uncertainty) remain unexamined. Future longitudinal studies tracking patients from pre-transplant evaluation to long-term recovery could elucidate how social support and resilience evolve across critical milestones (e.g., graft acceptance, relapse scares). Second, reliance on self-reported measures may introduce response bias, particularly for socially desirable constructs like resilience. For instance, participants might overreport familial harmony or underreport emotional distress due to cultural stigma. To enhance objectivity and mitigate this bias, future research should incorporate concrete triangulation strategies, such as clinician-rated assessments (e.g., structured psychiatric evaluations for anxiety/depression), objective behavioral metrics (e.g., immunosuppressant adherence monitored electronically), and biological stress markers (e.g., serial cortisol measurements). Third, the sample’s skewed demographics—75.2% male, predominantly younger adults—limit generalizability. Cultural norms emphasizing male authority in medical decisions (18) may explain this imbalance, but it also risks overlooking gender-specific challenges (e.g., caregiving burdens disproportionately affecting female recipients).

Future studies should employ stratified sampling to recruit underrepresented groups, including older adults (≥60 years) and women, and explore how gendered family roles modulate support effectiveness. Finally, while the study contextualizes resilience within Confucianism, it did not examine the critical influence of socioeconomic status (SES) and geographic origin (urban versus rural). These factors significantly impact both access to diverse forms of social support and the expression of cultural values. Regional heterogeneities in cultural practices (e.g., rural collectivism vs. urban individualism, ethnic minority traditions) combined with SES disparities were not examined. For example, rural families may rely more heavily on extended kinship networks for support, whereas higher-SES urban patients might prioritize professional counseling; similarly, economic constraints or urban/rural disparities in healthcare resources could profoundly affect stress levels and coping mechanisms. Future research should incorporate measures of SES and urbanicity to conduct comparative analyses, enabling the development of more nuanced and culturally adaptive interventions that resonate with localized values and resource availability.

### 4.4 Conclusion

This study identifies dual psychosocial pathways linking social support to QoL in Chinese heart transplant recipients: a direct path through enhanced familial cohesion and an indirect path through reduced illness uncertainty, with psychological resilience acting as a culturally significant moderator. Integrating Mishel’s Uncertainty in Illness Theory and the Connor-Davidson Resilience Scale within China’s collectivist context, we demonstrate how Confucian values, such as filial piety and collective endurance, shape adaptation processes, facilitating the management of uncertainty through family support. These findings address gaps in Western-centric psychosocial models by emphasizing cultural specificity, showing that resilience in this context functions not solely as individual trait but as a relational resource fostered within family narratives and communal obligations.

Clinically, these results highlight the need to develop family-integrated care protocols that combine culturally relevant resilience interventions (e.g., mindfulness integrated with family narratives) with structured family education to clarify post-transplant self-management. Policy initiatives should consider implementing routine psychosocial assessments to identify patients at higher risk due to low resilience or limited support networks, alongside exploring the potential for culturally adapted community-based peer support programs (e.g., tailored to rural kinship or urban professional settings). Integrating culturally informed, evidence-based psychosocial care within the Chinese transplant system provides a practical framework for improving long-term patient outcomes.

## Data Availability

The datasets analyzed during the current study contain sensitive information and are subject to ethical, legal, and privacy restrictions. Data may be made available by the corresponding author upon reasonable request with appropriate institutional review board (IRB) approval and data use agreements.
