# Supplementary material for: Social support and quality of life in Chinese heart transplant recipients: mediation through uncertainty in illness and moderation by psychological resilience
Source: Front Psychiatry. 2025 Jul 22;16:1637110. doi: 10.3389/fpsyt.2025.1637110 (PMC12338042; doi:10.3389/fpsyt.2025.1637110)
Supplement: Supplementary file 1 [file DataSheet1.docx]

Supplementary Materials

1. Details of measures
   1. Quality of Life

The Medical Outcomes Study Short Form-36 (SF-36), developed by the Boston Health Institute, was used to assess quality of life (Ware et al. 1993; Ware Jr 2000). This questionnaire comprises two domains: physical health (including Physical Functioning [PF], Role-Physical [RP], Bodily Pain [BP], and General Health [GH]) and mental health (including Vitality [VT], Social Functioning [SF], Role Emotional [RE], and Mental Health [MH]). The scale contains 36 items across 11 sections. The first item, which evaluates self-reported health changes, was excluded from scoring. Each dimension score was converted using the formula: Converted Score = (Raw Score − Minimum Possible Score) / (Maximum Possible Score − Minimum Possible Score) × 100. Total scores were calculated as the average of all eight dimensions. Physical and mental health sub-scores were derived by averaging their respective four dimensions, with higher scores indicating better quality of life. In this study, the Cronbach’s α coefficient for the SF-36 was 0.916.

- 1. Social support

Social support was evaluated using the SSRS developed by Xiao (1994). This 10-item self-report scale comprises three dimensions: objective support, subjective support, and support utilization. Items 1–4 and 8–10 were rated on a 4-point scale. Item 5 included five sub-items scored from 1 (no support) to 4 (full support). Items 6 and 7 were scored based on the number of support sources (0 = no sources). Total scores were summed across all items, with higher scores reflecting stronger social support. The Cronbach’s α coefficient for this measure was 0.92.

- 1. Uncertainty in illness

Uncertainty in illness was measured using the Chinese version of Mishel’s Uncertainty in Illness Scale (MUIS), which was translated and revised by Professor Xu Shulian (Xu and Huang 1996). This adapted scale consists of 33 items organized into four dimensions: Ambiguity (13 items: 3, 4, 8, 9, 13, 14, 16, 17, 18, 20, 23, 24, 26);Complexity (7 items: 6, 7, 10, 28, 31, 32, 33); Lack of Information (7 items: 1, 2, 5, 11, 19, 22, 29); Unpredictability (5 items: 12, 21, 25, 27, 30). Items were rated on a 5-point Likert scale ranging from:1 = Strongly disagree to 5 = Strongly agree. Twelve items (6, 7, 10, 12, 21, 25, 27, 28, 30, 31, 32, 33) required reverse scoring prior to analysis. Item 15 does not belong to any dimension and was excluded from the total score calculation. The total score range is 32 to 160, calculated by summing the scores of the 32 included items. Higher total scores indicate a greater level of illness-related uncertainty experienced by the patient. The Cronbach’s α coefficient in this study was 0.963.

- 1. Psychological resilience

Psychological Resilience was measured using the 25-item Connor-Davidson Resilience Scale (CD-RISC; Connor & Davidson, 2003), validated in Chinese populations(Yu, Sun, and Sun 2022). This scale comprises 25 items measuring resilience across three dimensions: Toughness, Strength, and Optimism. Responses were recorded on a 5-point Likert scale corresponding to the frequency of experiencing each item:0 = Never, 1 = Rarely, 2 = Sometimes, 3 = Often, 4 = Almost always. The total possible score ranges from 0 to 100. Higher total scores indicate a greater level of psychological resilience in the study participants. The Cronbach’s α coefficient for this scale was 0.948.

Reference

Ware, John E, Kristin K Snow, Mark Kosinski, and Barbara Gandek. 1993. "SF-36 health survey." *Manual and interpretation guide* 2.

Ware Jr, John E. 2000. "SF-36 health survey update." *Spine* 25 (24): 3130-3139.

Xiao, shuiyuan. 1994. "Theoretical foundation and research applications of the Social Support Rating Scale." *Journal of Clinical Psychiatry* 2: 98-100.

Xu, Shulian, and Xiuli Huang. 1996. "Mishel's Uncertainty in Illness Scale Chinese version test." *Chinese Nursing Research* 4 (1): 59-67.

Yu, Zhichao, Di Sun, and Jia Sun. 2022. "Social support and fear of cancer recurrence among Chinese breast cancer survivors: the mediation role of illness uncertainty." *Frontiers in psychology* 13: 864129.
